# Pyogenic Pericarditis Leading to Cardiac Arrest: a Case Report Highlighting POCUS-Guided Pericardiocentesis

**DOI:** 10.24908/pocusj.v11i01.19751

**Published:** 2026-04-22

**Authors:** Nadine Ajami, Brian Kohen, Donny Perez, Eric Boccio

**Affiliations:** 1 Memorial Healthcare System, Hollywood, FL, USA; 2 Mount Sinai Medical Center, Miami Beach, FL, USA

**Keywords:** Cardiac tamponade, Pyogenic pericarditis, Purulent effusion, Pericardiocentesis, Cardiac POCUS, Cardiac arrest, POCUS

## Abstract

A 44-year-old man with a history of intravenous drug use presented to the emergency department in cardiac arrest. Point of care ultrasound (POCUS) revealed a pericardial effusion with tamponade physiology. Initial landmark-guided pericardiocentesis returned blood but failed to confirm guidewire placement via POCUS. A repeat, ultrasound-guided pericardiocentesis yielded purulent fluid. Subsequent drainage of 50 mL of fluid improved cardiac contractility and hemodynamics. The patient was admitted to the intensive care unit, and pericardial fluid cultures grew *Streptococcus pneumoniae*. This case highlights the diagnostic and procedural utility of POCUS in managing purulent pericardial tamponade as a cause of cardiac arrest.

## Case presentation

**Figure 1.**
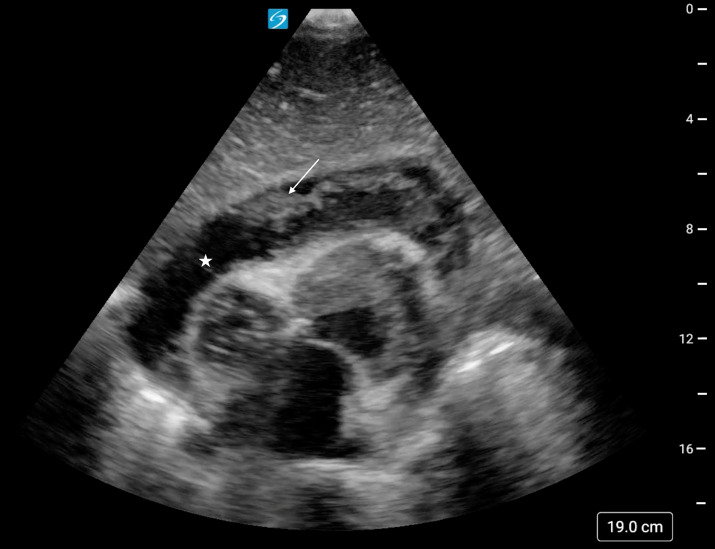
Subxiphoid cardiac point of care ultrasound (POCUS) view demonstrating a mixed echogenicity pericardial effusion (star) with fibrinous strands (arrow).

A 44-year-old man with a history of type I diabetes mellitus, cocaine abuse, and alcohol abuse presented to the emergency department (ED) in cardiac arrest after collapsing in the shower. The patient had been complaining of right upper quadrant abdominal pain radiating to the right shoulder for two weeks prior to the event. He received a total of 6 mg epinephrine, 1 g calcium gluconate, 50 mEq sodium bicarbonate, and 2 mg naloxone by emergency medical services (EMS). The patient was intubated prior to ED arrival. Endotracheal tube placement was confirmed prior to and after patient transfer from the EMS stretcher. Mechanical compressions were provided via a Lund University Cardiopulmonary Assist System (LUCAS) device. Upon initial pulse check, a carotid pulse was palpated and return of spontaneous circulation (ROSC) was noted. Initial post-ROSC vital signs were remarkable for hypothermia (33.3°C, axillary), tachycardia (127 beats per minute), and hypotension (66/40 mmHg). During resuscitation, cardiac point of care ultrasound (POCUS) examination was performed which revealed a pericardial effusion with concern for tamponade (Figure 1).

**Figure 2.**
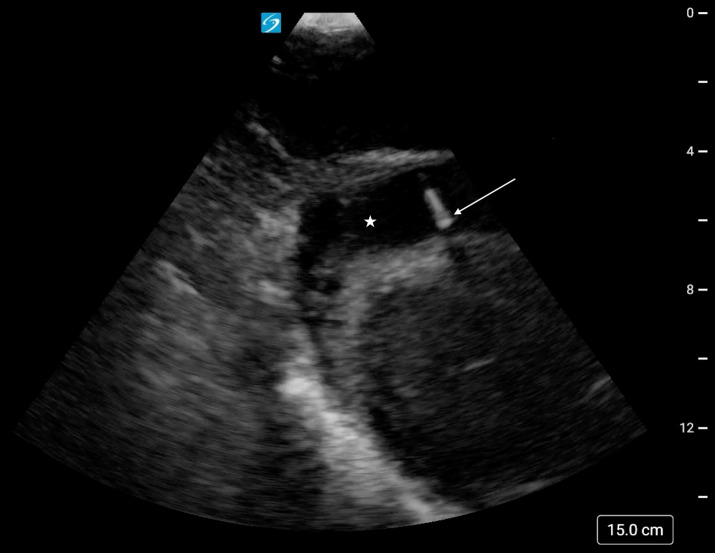
Subxiphoid cardiac point of care ultrasound (POCUS) view revealing the echogenic needle tip (arrow) positioned in the pericardial space (star) during the second attempt at pericardiocentesis.

Emergent pericardiocentesis was performed by interventional cardiology at bedside using a landmark approach with return of a scant volume of blood. Cardiac POCUS was employed by the emergency physician but failed to confirm visualization of the guidewire in the pericardial sac (Supplementary Material S1). The initial attempt was aborted, and pericardiocentesis was repeated under dynamic ultrasound guidance which yielded 50 mL of purulent fluid during aspiration (Figure 2). The pericardiocentesis needle tip was visualized using POCUS to be properly positioned within the pericardial sac (Supplementary Material S2).

**Figure 3.**
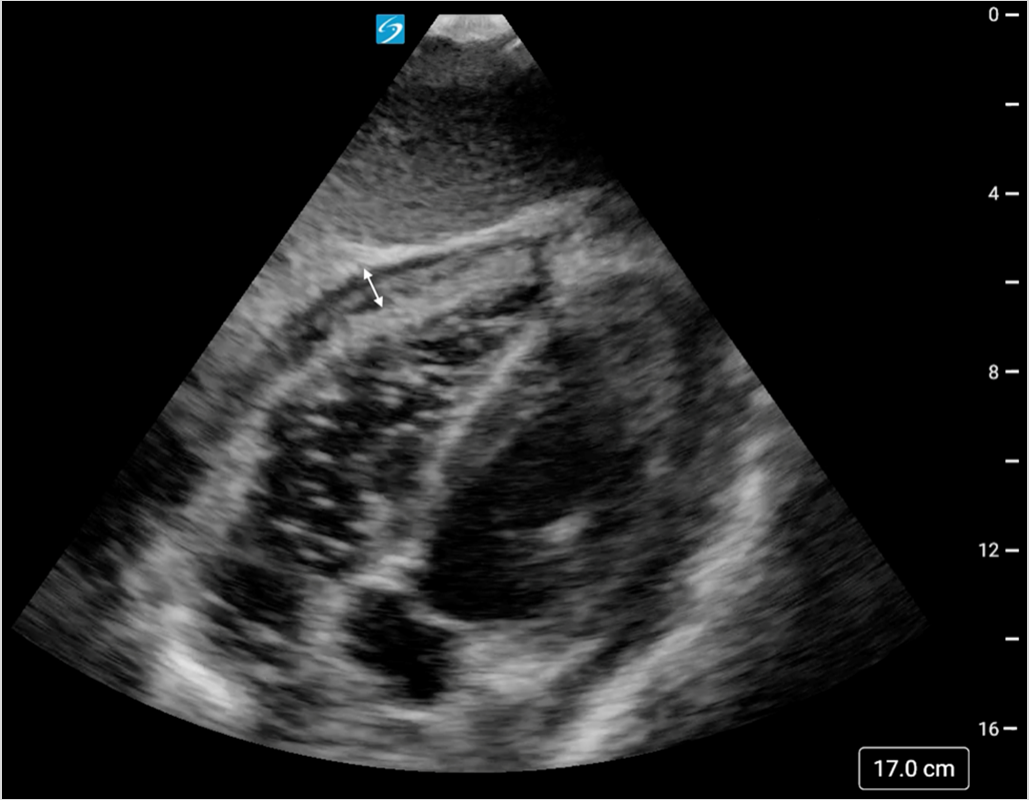
Subxiphoid cardiac point of care ultrasound (POCUS) view revealing a decrease in the size of the pericardial effusion (double-headed arrow) following aspiration of 50 mL of purulent fluid during the repeated attempt at pericardiocentesis under POCUS-guidance.

Repeat cardiac POCUS following aspiration of 50 mL of purulent fluid revealed a decrease in the volume of pericardial effusion and improved heart contractility which corresponded with an improvement in blood pressure to 128/106 mmHg (Figure 3, Supplementary Material S3).

**Figure 4.**
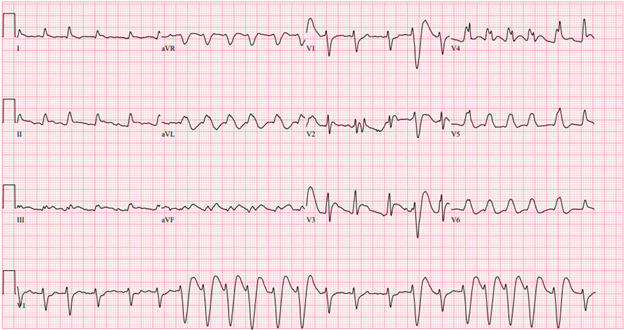
Initial emergency department electrocardiogram obtained post-return of spontaneous circulation demonstrating a wide complex sinus tachycardia.

Laboratory results revealed leukocytosis (white blood cell count, 42.5 × 103 cells/µL), hyperkalemia (6.0 mEq/ L), elevated serum creatinine (3.36 mg/ dL), hyperlactatemia (16.0 mmol/L), hyperglycemia (serum glucose, 554 mg/dL), and an anion gap (25 mEq/L). A chest X-ray revealed satisfactory endotracheal tube positioning, a right lower lobe infiltrate, small pericardial effusion, and a left-sided pleural effusion. The electrocardiogram (EKG) revealed wide complex tachycardia (Figure 4).

Empiric vancomycin and piperacillin/tazobactam were administered for septic shock presumed secondary to pyogenic pericarditis and community-acquired pneumonia. Given the clinical suspicion of diabetic ketoacidosis and hyperkalemia with EKG changes, an insulin infusion and continuous albuterol nebulization were initiated. The patient was admitted to the medical intensive care unit. Computed tomography of the chest revealed multifocal pneumonia and a moderate sized left hydropneumothorax. A left-sided thoracostomy with chest tube insertion was performed by the interventional pulmonology team. The pericardial fluid culture and subsequently collected blood cultures both grew *Streptococcus pneumoniae*. Following a “goals of care” discussion with the patient's family, the patient's code status was revised to Do Not Resuscitate, and the patient died on hospital day 4.

## Diagnosis

### Pericardial tamponade secondary to pyogenic pericarditis

Pyogenic pericarditis can rapidly progress to pericardial tamponade and precipitate cardiac arrest. The pathophysiology involves accumulation of pus within the pericardial space which increases intrapericardial pressure [1]. When this pressure exceeds the cardiac chambers' filling pressures, diastolic filling is impaired, leading to a drop in cardiac output and ultimately circulatory collapse [2]. Rapid accumulation is particularly dangerous because the pericardium cannot stretch quickly enough to accommodate the increased volume, resulting in a dramatic rise in intrapericardial pressure and acute hemodynamic compromise. This is in contrast to slowly accumulating effusions, where compensatory mechanisms may delay tamponade physiology [3,4]. In pyogenic effusions, the inflammatory process also causes pericardial thickening and may promote systemic illness, further exacerbating hemodynamic instability [1,2]. Clinically, patients present with hypotension, elevated jugular venous pressure, and muffled heart sounds (Beck's triad), which may rapidly progress to cardiac arrest if untreated [5–7]. The mortality rate is high, even with prompt intervention, due to the fulminant nature of infection and the risk of rapid decompensation.

## Discussion

Pyogenic pericarditis is a rare but potentially life-threatening condition due to its rapid progression to sepsis, pericardial tamponade, and hemodynamic collapse. The diagnosis is confirmed through culturing of pericardial fluid [8]. Pericardiocentesis may be performed using the landmark approach. However, POCUS-guided pericardiocentesis is recommended as it is associated with higher success rates and fewer complications [9]. In this case, the landmark approach was believed to have been successful due to aspiration of blood, however, proper placement of the needle could not be verified using POCUS. The second attempt at pericardiocentesis was successfully confirmed by real-time POCUS visualization of the needle in the pericardial space, aspiration of purulent fluid, improved hemodynamics, and a post-procedural reduction in the size of the pericardial effusion with reversal of radiographic tamponade on POCUS.

In the setting of cardiac arrest, the application of POCUS is essential for assessing cardiac motion, identifying potentially reversible etiologies, facilitating procedural guidance, and providing dynamic feedback on the efficacy of ongoing resuscitation efforts [10]. POCUS-guidance has become the standard of care for pericardiocentesis because it dramatically improves both safety and success rates compared to the blind landmark approach [11]. The most critical benefit is minimizing complications, as real-time visualization allows the proceduralist to avoid vital surrounding structures such as the lungs, internal thoracic vessels, and cardiac chambers. Furthermore, ultrasound excels at optimizing the insertion site by locating the area with the maximal fluid accumulation, ensuring the distance from the skin to the fluid is the shortest [12]. While the subxiphoid approach is classic, POCUS guidance allows for the safe use of alternative approaches such as the apical and parasternal when they offer more direct access to a non-uniform fluid collection. Real-time procedure monitoring is also a key advantage, as continuous visualization confirms the correct path of the needle and proper catheter placement in the pericardial space [13].

Needle and catheter placement during pericardiocentesis may be confirmed through a combination of aspiration, diagnostic imaging, and physiological reassessment. The first sign of correct needle placement is the ability to freely aspirate pericardial fluid into the syringe, often achieved after advancing the needle 1–2 mm past an initial partial entry where aspiration was intermittent. Once a guidewire is advanced, confidence regarding correct positioning in the pericardial space is achieved if the wire appears to span multiple cardiac chambers on imaging. Proceduralists must remain vigilant for signs of cardiac puncture, which include movement of the needle with each cardiac cycle, the presence of premature ventricular complexes on telemetry, brisk blood return from the needle lumen, flailing movement of the wire during systole, or wire tracking along the pulmonary artery or aorta. After the needle is exchanged for a catheter, correct placement is confirmed using two primary methods: transducing a non-ventricular pressure waveform on a monitor and confirmation on imaging. When using POCUS, placement confirmation involves injecting agitated saline bubbles into the catheter and then visualizing them in the pericardial space. Bubbles in the atria or ventricles are highly suggestive of cardiac puncture and ectopic catheter placement. In very large or loculated effusions, inadequate bubble visualization may necessitate the use of additional imaging views for accurate confirmation [14].

Pericardial tamponade secondary to pyogenic pericarditis is a true cardiac emergency with a high risk of death if it is not managed expeditiously. Immediate drainage of the pericardial effusion and administration of broad-spectrum antibiotics are essential to improve hemodynamics and treat the underlying infection. When performing emergent pericardiocentesis, POCUS-guidance is recommended over the landmark technique, as it provides real-time visual confirmation of proper needle, guidewire, and catheter positioning thereby increasing procedural success rates and reducing complications related to ectopic needle placement.

## References

[1] Patel H, Patel C, Soni M, Patel A, Banda V. Acute Primary Pneumococcal Purulent Pericarditis With Cardiac Tamponade: A Case Report and Literature Review. Medicine (Baltimore). 2015;94(41):e1709. doi: 10.1097/MD.0000000000001709 26469910 PMC4616809

[2] Zmora I, Wiener-Well Y, Alpert EA. A case of purulent pneumococcal pericarditis. Am J Emerg Med. 2019;37(5):1006.e5–1006.e7. doi: 10.1016/j.ajem.2019.02.013 30777376

[3] Adler Y, Ristić AD, Imazio M, Brucato A, Pankuweit S, Burazor I, Seferović PM, Oh JK. Cardiac tamponade. Nat Rev Dis Primers. 2023;9(1):36. doi: 10.1038/s41572-023-00446-1 37474539

[4] Cremer PC, Klein AL, Imazio M. Diagnosis, Risk Stratification, and Treatment of Pericarditis: A Review. JAMA. 2024;332(13):1090–1100. doi: 10.1001/jama.2024.12935 39235771

[5] Troughton RW, Asher CR, Klein AL. Pericarditis. Lancet. 2004;363(9410):717–27. doi: 10.1016/S0140-6736(04)15648-1 15001332

[6] Roy CL, Minor MA, Brookhart MA, Choudhry NK. Does this patient with a pericardial effusion have cardiac tamponade? JAMA. 2007;297(16):1810–8. doi: 10.1001/jama.297.16.1810 17456823

[7] Bodson L, Bouferrache K, Vieillard-Baron A. Cardiac tamponade. Curr Opin Crit Care. 2011;17(5):416–24. doi: 10.1097/MCC.0b013e3283491f27 21716107

[8] Adler Y, Charron P, Imazio M, Badano L, Barón-Esquivias G, Bogaert J, Brucato A, Gueret P, Klingel K, Lionis C, Maisch B, Mayosi B, Pavie A, Ristic AD, Sabaté Tenas M, Seferovic P, Swedberg K, Tomkowski W; ESC Scientific Document Group. 2015 ESC Guidelines for the diagnosis and management of pericardial diseases: The Task Force for the Diagnosis and Management of Pericardial Diseases of the European Society of Cardiology (ESC)Endorsed by: The European Association for Cardio-Thoracic Surgery (EACTS). Eur Heart J. 2015;36(42):2921–2964. doi: 10.1093/eurheartj/ehv318 26320112 PMC7539677

[9] Blanco P, Figueroa L, Menéndez MF, Berrueta B. Pericardiocentesis: ultrasound guidance is essential. Ultrasound J. 2022;14(1):9. doi: 10.1186/s13089-022-00259-5 35157176 PMC8844339

[10] Magon F, Longhitano Y, Savioli G, Piccioni A, Tesauro M, Del Duca F, Napoletano G, Volonnino G, Maiese A, La Russa R, Di Paolo M, Zanza C. Point-of-Care Ultrasound (POCUS) in Adult Cardiac Arrest: Clinical Review. Diagnostics (Basel). 2024;14(4):434. doi: 10.3390/diagnostics14040434 38396471 PMC10887671

[11] Salem K, Mulji A, Lonn E. Echocardiographically guided pericardiocentesis - the gold standard for the management of pericardial effusion and cardiac tamponade. Can J Cardiol. 1999;15(11):1251–5 10579740

[12] Callahan JA, Seward JB, Tajik AJ. Cardiac tamponade: pericardiocentesis directed by two-dimensional echocardiography. Mayo Clin Proc. 1985;60(5):344–7. doi: 10.1016/s0025-6196(12)60541-2 3990381

[13] Tsang TS, Enriquez-Sarano M, Freeman WK, Barnes ME, Sinak LJ, Gersh BJ, Bailey KR, Seward JB. Consecutive 1127 therapeutic echocardiographically guided pericardiocenteses: clinical profile, practice patterns, and outcomes spanning 21 years. Mayo Clin Proc. 2002;77(5):429–36. doi: 10.4065/77.5.429 12004992

[14] Tsang TS, Freeman WK, Sinak LJ, Seward JB. Echocardiographically guided pericardiocentesis: evolution and state-of-the-art technique. Mayo Clin Proc. 1998;73(7):647–52. doi: 10.1016/S0025-6196(11)64888-X 9663193

